# TB and poverty: the effect of rifampicin-resistant TB on household income

**DOI:** 10.5588/ijtldopen.23.0607

**Published:** 2024-04-01

**Authors:** A. Ciobanu, V. Plesca, S. Doltu, M. Manea, L. Domente, A. Dadu

**Affiliations:** ^1^ Health Primary Care Department, University of Medicine and Pharmacy “N. Testemitanu”, Chisinau, Republic of Moldova;; ^2^ WHO Regional Office for Europe, Copenhagen, Denmark;; ^3^ Institute of Phthisiopneumology “Chiril Draganiuc”, Chisinau,; ^4^ National Agency of Public Health, Chisinau,; ^5^ Act for Involvement (nongovernmental organization), Chisinau,; ^6^ Independent Consultant, Chisinau, Republic of Moldova

**Keywords:** rifampicin-resistant tuberculosis, Republic of Moldova, income, RR-TB, out-of-pocket payment

## Abstract

**SETTING:**

The Republic of Moldova, one of Europe's poorest countries, also bears one of the highest burdens of rifampicin-resistant TB (RR-TB).

**OBJECTIVES:**

To trace the patients’ journey through TB in terms of the relationship with poverty and assess its determinants.

**DESIGN:**

This cross-sectional study used secondary data from a survey assessing catastrophic costs in RR-TB-affected households.

**RESULTS:**

Data were obtained from 430 RR-TB patients. The percentage of poor TB-affected households rose from 65% prior to TB to 86% after TB treatment completion (*P* < 0.001). Social factors leading to poverty were identified for each stage: diagnostic period (history of incarceration: cOR 2.3, 95% CI 1.1–5.2); treatment period (being unemployed or unofficially employed: cOR 6.7, 95% CI 4.3–10.0); and post-treatment (being married or cohabiting: cOR 5.7, 95% CI 2.9–11.0). Participants who had ≥3 members in their households were more likely to be poor at all TB stages: diagnostic period (cOR 5.7, 95% CI 3.7–8.8), treatment period (cOR 3.8, 95% CI 2.5–5.6) and post-treatment (cOR 7.2, 95% CI 3.6–14.3).

**CONCLUSION:**

The study identified risk factors associated with poverty at each stage of TB. These findings outline that innovative social protection policies are required to protect TB patients against poverty.

## RÉSUMÉ

**CONTEXTE:**

La République de Moldavie est l'un des pays les plus pauvres d'Europe et l'un des plus touchés par la TB résistante à la rifampicine (RR-TB).

**OBJECTIFS:**

Nous avons cartographié le parcours des patients atteints de TB en lien avec la pauvreté et évalué les déterminants associés.

**MÉTHODE:**

Cette étude transversale a analysé des données secondaires issues d'une enquête évaluant les coûts catastrophiques supportés par les ménages touchés par la RR-TB.

**RÉSULTATS:**

Des données ont été recueillies auprès de 430 patients atteints de RR-TB. Le taux de ménages pauvres touchés par la TB est passé de 65% avant le traitement à 86% après la fin du traitement de la TB (*P* < 0,001). Pour chaque stade de la TB, les facteurs sociaux conduisant à la pauvreté ont été identifiés : période de diagnostic (antécédents d'emprisonnement : rapport de cotes brut (cOR) 2,3, IC à 95% 1,1–5,2) ; période de traitement (être au chômage ou employé officieux : cOR 6,7 ; IC 95% 4,3–10,0) ; et post-traitement (être marié ou cohabitant : cOR 5,7, IC 95% 2,9–11,0). Les participants dont le ménage comptait ≥3 membres étaient plus susceptibles d'être pauvres à tous les stades de la TB : période de diagnostic (cOR 5,7 ; IC à 95% 3,7–8,8), période de traitement (cOR 3,8 ; IC à 95% 2,5–5,6) et post-traitement (cOR 7,2 ; IC à 95% 3,6–14,3).

**CONCLUSION:**

L'étude a permis d'identifier des facteurs de risque liés à la pauvreté à toutes les étapes de la TB. Ces résultats soulignent l’importance de mettre en place des politiques de protection sociale novatrices pour prévenir l'appauvrissement des patients atteints de TB.

Equity in access to healthcare is today’s greatest health challenge, and it will be achieved by accelerating the progress in healthcare provision to poor and socially vulnerable groups.^1^ There is a strong link between poverty and health,^2^ and poverty and TB have a symbiotic relationship. The WHO’s Global TB report provides substantial evidence that poverty is an enabler of TB,^3^ and there is a strong association between gross domestic product (GDP) per capita and TB incidence.^4^ Reducing poverty, advocating improved equity of access to health care and universal health care are key components of WHO’s End TB Strategy 2016–2035.^5^

The Republic of Moldova is a lower-middle-income country where the poverty headcount ratio at national poverty lines is 23% of the population.^6^ The gross annual national income in the country is US$3,930 per capita.^7^ The Republic of Moldova is one of the 18 high-priority countries for TB of the WHO European Region,^8^ and one of the 30 countries with the highest burdens of rifampicin-resistant TB (RR-TB) cases in the world, despite managing, since 2007, to reverse the rising TB incidence rate. In recent years, the TB incidence rate has continued to decline by an average of 3.2% per year.^3,8^ The successful treatment of RR-TB cases was 53% in 2016.^8^

Direct TB investigations and all TB treatment are free of charge in the country.^9^ However, despite this, patients can face catastrophic health expenditures because of the high costs of diagnostic period care and the high indirect costs incurred during treatment. The survey conducted in 2016 showed that catastrophic costs were experienced by 26% of households affected by RR-TB, and also found that patients with RR-TB lost 30% of their income in inpatient care and 70% in outpatient care.^10^

Understanding the bidirectional relationship between TB and poverty would be a powerful step towards breaking this vicious cycle and reducing the TB burden. This study aimed to determine the proportion of households affected by RR-TB who experienced poverty during the diagnostic period, during treatment and after the completion of TB treatment, and to assess the risk factors associated with poverty at each TB stage. The study findings will allow decision-makers to identify solutions and interventions for both financial and social protection to reduce poverty, and to improve TB control in the country.

## METHODS

### Study design

This is cross-sectional study using secondary data from patients with RR-TB identified in 2016 survey on catastrophic costs carried out in the Republic of Moldova.

### General setting

The Republic of Moldova is situated in south-eastern Europe and has a population at about 3 million inhabitants (including about 0.5 million inhabitants in the breakaway region of the Left Bank of the River Dniester).

### National tuberculosis control

The Ministry of Health is the body primarily responsible for TB control in the country. The National Tuberculosis Control Programme (NTP) was initiated in 2001 and has been approved every 5 years since its inception. TB patients receive treatment in both inpatient and outpatient care; in 2016, approximately 90% of patients with RR-TB were hospitalised for an average of 5 months. Direct TB investigations and all TB medicines are free of charge in the country. Health insurance coverage in the general population is 70–75%,^11^ but in TB patients, this is 30–35%.^12^ The lack of health insurance can mean that it is difficult for TB patients to access medical services before their TB diagnosis has been established and to receive medications for side-effects in special outpatient care. Temporary disability allowances and adherence incentives for the entire period of treatment are available for TB patients as welfare benefits. Temporary disability allowances are granted for those who were officially employed before being diagnosed with TB and cover 100% of wages during the entire period of treatment, and incentives are given monthly for taking 90% of doses given in outpatient care. Two types of incentive amounts were paid during the study period: US$20 per month in the period before 1 July 2015 and US$56 per month in the period after 1 July 2015. There is a national TB case-based database – Information System for Monitoring and Evaluation TB patients (SIME TB) – for registering TB patients and follow-up.^12^

### Study population

The study population in the baseline study^10^ included 430 RR-TB patients with the following inclusion criteria: new or relapsed RR-TB cases; adult (≥18 years); receiving treatment for the intensive phase of TB treatment in hospital for at least 2 months, or receiving treatment for the continuation phase of treatment as outpatients for at least 2 months, or having completed treatment for at least 2 months before interview. Patients from prisons and the Left Bank of the River Dniester were excluded. In this study, the term ‘household’ is defined as a group of relatives or non-relatives dwelling under one roof and sharing one budget, or a person living alone and self-managing who does not belong to any other household.

### Data collection, sources and variables

The stages of TB were defined as the diagnostic period (2 months before TB diagnosis), treatment period (from the beginning of treatment to the end of treatment) and the post-treatment period (2 months after treatment completion). The procedures for data collection from the questionnaires are described in the original study.^10^ Data extracted from the original study were social and demographic variables (sex, age, residence, education, marital status, employment, household size, health insurance, history of labour migration and incarceration, smoking status, alcohol consumption, drug use), clinical variables (history of TB treatment, presence of HIV coinfection, presence of diabetes mellitus, presence of comorbidities and presence of side effects during TB treatment), sources of finance used to pay for out-of-pocket (OOP) expenditure (savings, sale of possessions, uptake of loans and income) and household income. Each respondent was interviewed just once, reporting on TB-related costs for the specific phase he/she was interviewed for. To measure the income for each household, we established the income earned by patients and other family members from wages, welfare benefits, adherence incentives and other income sources. To estimate the entire household income, we extrapolated the monthly reported income for the diagnostic period, the treatment period and the post-treatment period for all study participants. Using the World Bank’s poverty line standard of US$1.90 per person per day,^13^ we grouped households into poor (earning ≤US$1.9 per capita per day) and non-poor (earning >US$1.9 per capita per day) households.

### Data analysis

Extracted data (from the original study) were imported into SPSS^®^ Statistics v20.0 (IBM, Armonk, NY, USA) for analyses. The data were described using frequency, proportions, mean (standard deviation [SD]), and median (interquartile range [IQR]). The χ^2^ test was used to assess patients’ profiles and to compare proportions, and *t*-tests were used for continuous variables. If the expected cell frequency was less than 5, Fisher’s Exact test was used instead of the χ^2^ test. The Kruskal–Wallis test was used to compare medians of incomes between groups. The crude odds ratio (cOR) was selected as a measure of association in the analysis of poverty determinants. We used the RawGraphs web-application to construct an alluvial plot to visualise the change in household poverty at the diagnostic period, treatment period and post-treatment stages of TB.^14^ Cochran’s *Q* test was selected to determine the differences of poverty levels between TB stages. For all analyses, levels of significance were set at 5%.

### Ethical approval

The study protocol was approved by the National Committee for Ethical Expertise of Clinical Trial, Chisinau, Republic of Moldova. Written informed consent was obtained from all participants of the baseline study.

## RESULTS

Demographic and clinical characteristics of study participants are shown in the Table. Of the 430 participants, 336 (78%) were male and 94 (22%) were female with an average age of 42 years (SD ±12.4); 150 participants were inpatients in the intensive phase of treatment, 137 patients were outpatients in the continuation phase of treatment and 143 participants had completed treatment. Most participants were from rural areas (63%), were unofficially employed or unemployed (62%) and had no health insurance (63%) (Table).

**Table.** **A)** Sociodemographic and clinical characteristics of poor and non-poor patients with RR-TB during the TB diagnostic period and other factors contributing to poverty.*

Characteristics	Total	Poor	Non-poor	*P*-value	OR (95%CI)
Total		280 (65.1)	150 (34.9)		
Sex					
Male	336 (78.1)	214 (63.7)	122 (36.3)	0.271	0.7 (0.4–1.2)
Female	94 (21.9)	66 (70.2)	28 (29.8)		Ref
Age, years					
≤39	198 (46.0)	129 (65.2)	69 (34.8)	0.989	1.0 (0.7–1.5)
≥40	232 (54.0)	151 (65.1)	81 (34.9)		Ref
Residence					
Urban	160 (37.2)	98 (61.3)	62 (38.8)	0.195	Ref
Rural	270 (62.8)	182 (67.4)	88 (32.6)		1.3 (0.9–2.0)
Level of education					
No/primary	143 (33.3)	99 (69.2)	44 (30.8)	0.234	1.4 (0.8–2.6)
Secondary	215 (50.0)	137 (63.7)	78 (36.3)	0.691	1.1 (0.6–1.9)
Higher	72 (16.7)	44 (61.1)	28 (38.9)		Ref
Marital status					
Married/cohabitation	224 (52.1)	155 (69.2)	69 (30.8)	0.064	1.4 (1.0–2.2)
Unmarried	206 (47.9)	125 (60.7)	81 (39.3)		Ref
Household size, person					
1 or 2	195 (45.3)	87 (44.6)	108 (55.4)	<0.001	Ref
>3	235 (54.7)	193 (82.1)	42 (17.9)		5.7 (3.7–8.8)
Employment					
Official employed	162 (37.7)	102 (63.0)	60 (37.0)	0.466	Ref
Unofficially employed or unemployed	268 (62.3)	178 (66.4)	90 (33.6)		1.2 (0.8–1.7)
Health insurance^†^					
Yes	159 (37.2)	141 (62.4)	85 (37.6)	0.212	Ref
No	268 (62.8)	139 (68.1)	65 (31.9)		1.3 (0.9–1.9)
History of labour migration					
Yes	78 (18.1)	37 (47.4)	41 (52.6)	<0.001	0.4 (0.2–0.7)
No	352 (81.9)	243 (69.0)	109 (31.0)		Ref
History of incarceration^‡^					
Yes	41 (9.8)	33 (80.5)	8 (19.5)	0.038	2.3 (1.1–5.2)
No	378 (90.2)	241 (63.8)	137 (36.2)		Ref
Current smoking status					
Yes	246 (57.2)	153 (62.2)	93 (37.8)	0.142	0.7 (0.5–1.1)
No	184 (42.8)	127 (69.0)	57 (31.0)		Ref
Alcohol consumption					
Yes	45 (10.5)	28 (62.2)	17 (37.8)	0.741	0.9 (0.5–1.6)
No	385 (89.5)	252 (65.5)	133 (34.5)		Ref
Injectable/non-injectable drug use					
Yes	43 (10.0)	29 (67.4)	14 (32.6)	0.866	1.1 (0.6–2.2)
No	387 (90.0)	251 (64.9)	136 (35.1)		0.9 (0.6–1.4)
History of TB treatment					
New	289 (67.2)	190 (65.7)	99 (34.3)	0.696	Ref
Relapsed	141 (32.8)	90 (63.8)	51 (36.2)		
HIV					
Yes	36 (8.4)	19 (52.8)	17 (47.2)	0.143	1.8 (0.8–3.7)
No	394 (91.6)	261 (66.2)	133 (33.8)		Ref
Diabetes mellitus					
Yes	11 (2.6)	8 (72.7)	3 (27.3)	0.754	0.7 (0.1–2.9)
No	419 (97.4)	272 (64.9)	147 (35.1)		Ref
Comorbidities					
Yes	135 (31.4)	83 (61.5)	52 (38.5)	0.287	1.3 (0.8–1.9)
No	295 (68.6)	197 (66.8)	98 (33.2)		Ref
Side effects					
Yes	352 (81.9)	230 (65.3)	122 (34.7)	0.898	1.1 (0.6–1.8)
No	78 (18.1)	50 (64.1)			Ref

**Table:** **B)** Sociodemographic and clinical characteristics of poor and non-poor patients with RR-TB during the treatment period and other factors contributing to poverty.*

Characteristics	Total	Poor	Non-poor	*P*-value	OR (95%CI)
Total		238 (55.3)	192 (44.7)		
Sex					
Male	336 (78.1)	184 (54.8)	152 (45.2)	0.643	0.9 (0.6–1.4)
Female	94 (21.9)	54 (57.4)	40 (42.6)		Ref
Age, years					
≤39	198 (46.0)	118 (59.6)	80 (40.4)	0.102	1.4 (0.9–2.0)
≥40	232 (54.0)	120 (51.7)	112 (48.3)		Ref
Residence					
Urban	160 (37.2)	88 (55.0)	72 (45.0)		Ref
Rural	270 (62.8)	150 (55.6)	120 (44.4)	0.911	1.0 (0.7–1.5)
Level of education					
No/primary	143 (33.3)	85 (59.4)	58 (40.6)	0.089	1.6 (0.9–2.9)
Secondary	215 (50.0)	119 (55.3)	96 (44.7)	0.232	1.4 (0.8–2.4)
Higher	72 (16.7)	34 (47.2)	38 (52.8)		Ref
Marital status					
Married/cohabitation	224 (52.1)	127 (56.7)	97 (43.3)	0.558	
Unmarried	206 (47.9)	111 (53.9)	95 (46.1)		Ref
Household size, person					
1 or 2	195 (45.3)	74 (37.9)	121 (62.1)		Ref
>3	235 (54.7)	164 (69.8)	71 (30.2)	<0.001	3.8 (2.5–5.6)
Employment					
Official employed	162 (37.7)	45 (27.8)	117 (72.2)	<0.001	Ref
Unofficially employed or unemployed	268 (62.3)	193 (72.0)	75 (28.0)		6.7 (4.3–10)
Health insurance^†^					
Yes	159 (37.2)	79 (49.7)	80 (50.3)		Ref
No	268 (62.8)	157 (58.6)	111 (41.4)	0.037	1.4 (1.0–2.1)
History of labour migration					
Yes	78 (18.1)	42 (53.8)	36 (46.2)	0.768	0.9 (0.6–1.5)
No	352 (81.9)	196 (55.7)	156 (44.3)		Ref
History of incarceration^‡^					
Yes	41 (9.8)	24 (58.5)	17 (41.5)	0.742	1.1 (0.6–2.2)
No	378 (90.2)	208 (55.0)	170 (45.0)		Ref
Current smoking status					
Yes	246 (57.2)	132 (53.7)	114 (46.3)	0.415	0.8 (0.6–1.2)
No	184 (42.8)	106 (57.6)	78 (42.4)		Ref
Alcohol consumption					
Yes	45 (10.5)	26 (57.8)	19 (42.2)	0.855	1.1 (0.6–2.2)
No	385 (89.5)	212 (55.1)	173 (44.9)		Ref
Injectable/non-injectable drug use					
Yes	43 (10.0)	19 (44.2)	24 (55.8)	0.146	0.6 (0.3–1.1)
No	387 (90.0)	219 (56.6)	168 (43.4)		Ref
History of TB treatment					
New	289 (67.2)	165 (57.1)	124 (42.9)		Ref
Relapsed	141 (32.8)	73 (51.8)	68 (48.2)	1.150	0.8 (0.6–1.2)
HIV					
Yes	36 (8.4)	22 (61.1)	14 (38.9)	0.490	1.3 (0.6–2.7)
No	394 (91.6)	216 (54.8)	178 (45.2)		Ref
Diabetes mellitus					
Yes	11 (2.6)	4 (36.4)	7 (63.6)	0.329	0.4 (0.1–1.8)
No	419 (97.4)	234 (55.8)	185 (44.2)		Ref
Comorbidities					
Yes	135 (31.4)	75 (55.6)	60 (44.4)	0.953	1.0 (0.7–1.5)
No	295 (68.6)	163 (55.3)	132 (44.7)		Ref
Side effects					
Yes	352 (81.9)	188 (53.4)	164 (46.6)	0.086	0.6 (0.4–1.1)
No	78 (18.1)	50 (64.1)	28 (35.9)		Ref

**Table:** **C)** Sociodemographic and clinical characteristics of poor and non-poor patients with RR-TB during the post-treatment period and other factors contributing to poverty.*

Characteristics	Total	Poor	Non-poor	*P*-value	OR (95%CI)
Total		368 (85.6)	62 (14.4)		
Sex					
Male	336 (78.1)	285 (84.8)	51 (15.2)	0.506	0.7 (0.4–1.5)
Female	94 (21.9)	83 (88.3)	11 (11.7)		Ref
Age, years					
≤39	198 (46.0)	175 (88.4)	23 (11.6)	0.126	1.5 (0.9–2.7)
≥40	232 (54.0)	193 (83.2)	39 (16.8)		Ref
Residence					
Urban	160 (37.2)	140 (87.5)	20 (12.5)		Ref
Rural	270 (62.8)	228 (84.4)	42 (15.6)	0.399	0.8 (0.4–1.4)
Level of education					
No/primary	143 (33.3)	115 (80.4)	28 (19.6)	1.000	0.5 (0.2–1.2)
Secondary	215 (50.0)	189 (87.9)	26 (12.1)	0.163	0.9 (0.4–2.1)
Higher	72 (16.7)	64 (88.9)	8 (11.1)		Ref
Marital status					
Married/cohabitation	224 (52.1)	212 (94.6)	12 (5.4)	<0.001	5.7 (2.9–11.0)
Unmarried	206 (47.9)	156 (75.7)	50 (24.3)		Ref
Household size, person					
1 or 2	195 (45.3)	144 (73.8)	51 (26.2)		Ref
>3	235 (54.7)	224 (95.3)	11 (4.7)	<0.001	7.2 (3.6–14.3)
Employment					
Official employed	162 (37.7)	138 (85.2)	24 (14.8)		Ref
Unofficially employed or unemployed	268 (62.3)	230 (85.8)	38 (14.2)	0.888	1.0 (0.6–1.8)
Health insurance^†^					
Yes	159 (37.2)	195 (86.3)	31 (13.7)		Ref
No	268 (62.8)	173 (84.8)	31 (15.2)	0.663	0.9 (0.5–1.5)
History of labour migration					
Yes	78 (18.1)	71 (91.0)	7 (9.0)	0.155	1.9 (0.8–4.3)
No	352 (81.9)	297 (84.4)	55 (15.6)		Ref
History of incarceration^‡^					
Yes	41 (9.8)	35 (85.4)	6 (14.6)		1.0 (0.4–2.4)
No	378 (90.2)	324 (85.7)	54 (14.3)	1.000	Ref
Current smoking status					
Yes	246 (57.2)	208 (84.6)	38 (15.4)	0.579	0.8 (0.5–1.4)
No	184 (42.8)	160 (87.0)	24 (13.0)		Ref
Alcohol consumption					
Yes	45 (10.5)	38 (84.4)	7 (15.6)	0.961	0.9 (0.4–2.1)
No	385 (89.5)	330 (85.7)	55 (14.3)		Ref
Injectable/non-injectable drug use					
Yes	43 (10.0)	38 (88.4)	5 (11.6)	0.819	1.3 (0.5–3.5)
No	387 (90.0)	330 (85.3)	57 (14.7)		Ref
History of TB treatment					
New	289 (67.2)	251 (86.9)	38 (13.1)		Ref
Relapsed	141 (32.8)	117 (83.0)	24 (17.0)	0.353	0.7 (0.4–1.3)
HIV					
Yes	36 (8.4)	32 (88.9)	4 (11.1)	0.804	1.4 (0.5–5.5)
No	394 (91.6)	336 (85.3)	58 (14.7)		Ref
Diabetes mellitus					
Yes	11 (2.6)	7 (63.6)	4 (36.4)	0.059	0.3 (0.1–1.0)
No	419 (97.4)	361 (86.2)	58 (13.8)		Ref
Comorbidities					
Yes	135 (31.4)	110 (81.5)	25 (18.5)	0.140	0.6 (0.4–1.1)
No	295 (68.6)	258 (87.5)	37 (12.5)		Ref
Side effects					
Yes	352 (81.9)	301 (85.5)	51 (14.5)	1.000	1.0 (0.5–1.9)
No	78 (18.1)	67 (85.9)	11 (14.1)		Ref

* Poverty defined according to World Bank criterion and based on household income prior to TB diagnosis. For statistical analysis, χ^2^ tests were used unless expected cell frequency was less than 5 in which case Fisher’s exact test was used. Missing data were excluded during hypothesis testing.

† Data available from 427 patients.

‡ Data available from 419 patients.

RR-TB = rifampicin-resistant TB; OR = odds ratio; CI = confidence interval; ref = reference.

**Figure 1.**
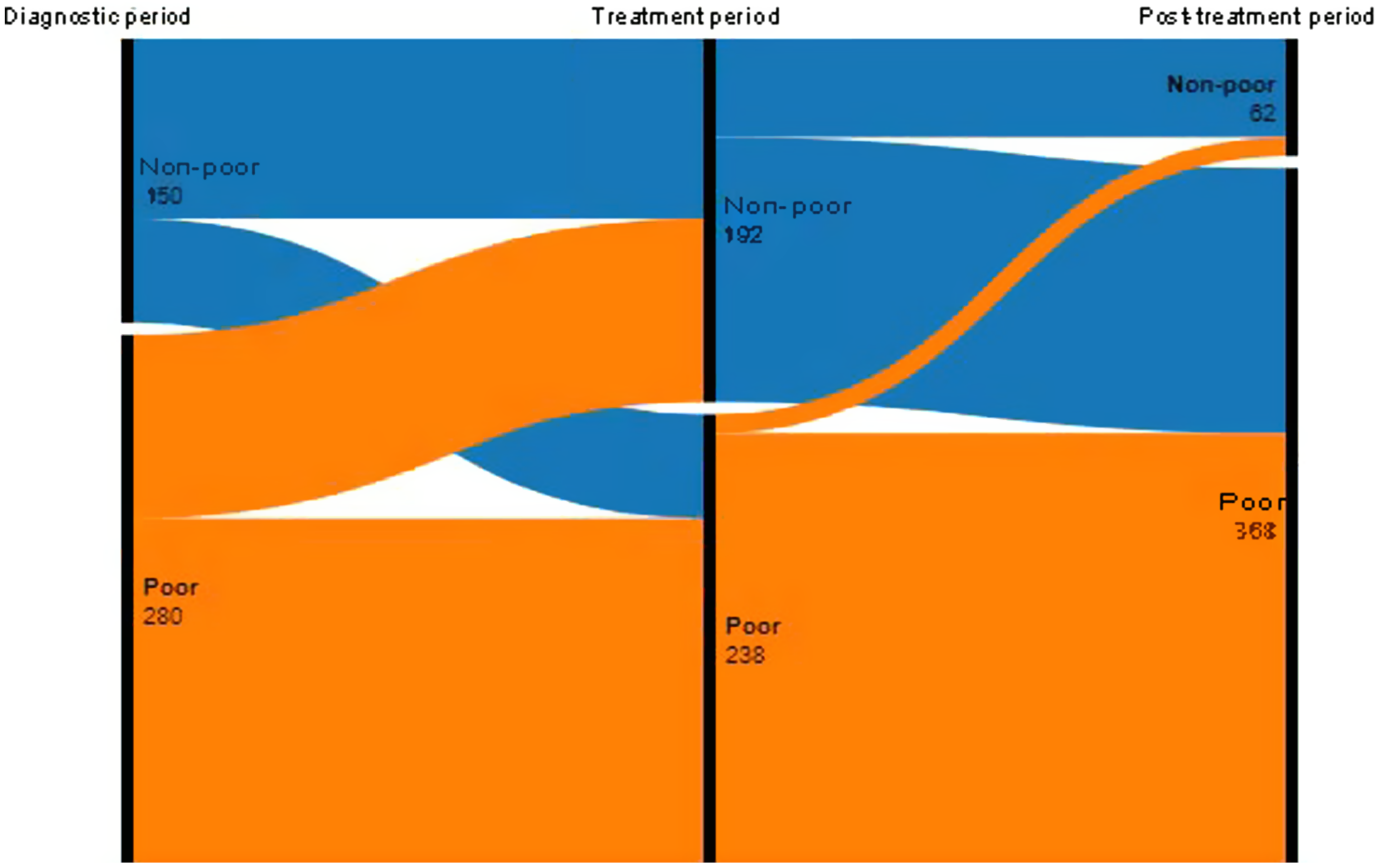
Change in household income over time according to TB treatment stage for patients with RR-TB. Alluvial plot representing economic changes of poor (orange) and non-poor (blue) patients with RR-TB during TB stages from before being diagnosed with TB to after TB treatment completion. The three bars represent the TB stages: diagnostic period, treatment period and post-treatment period. Comparison between three groups (diagnostic period, treatment period, post-treatment) *P* < 0.001 using the Cochran test. RR-TB = rifampicin-resistant TB.

We measured household poverty at each stage of TB. In this study, 65% (280/430) of households were poor prior to TB and 86% were poor (368/430) after completion of TB treatment. More than a third (55/150, 37%) of those who were non-poor before TB became poor during TB treatment. After treatment completion, 73% (140/192) of those who were non-poor during TB treatment became poor, and only 4% (10/238) of those who were poor during TB treatment became non-poor after treatment completion. Our analysis showed a strong relationship between poverty and TB pathways (*P* < 0.001) (Figure 1).

**Figure 2.**
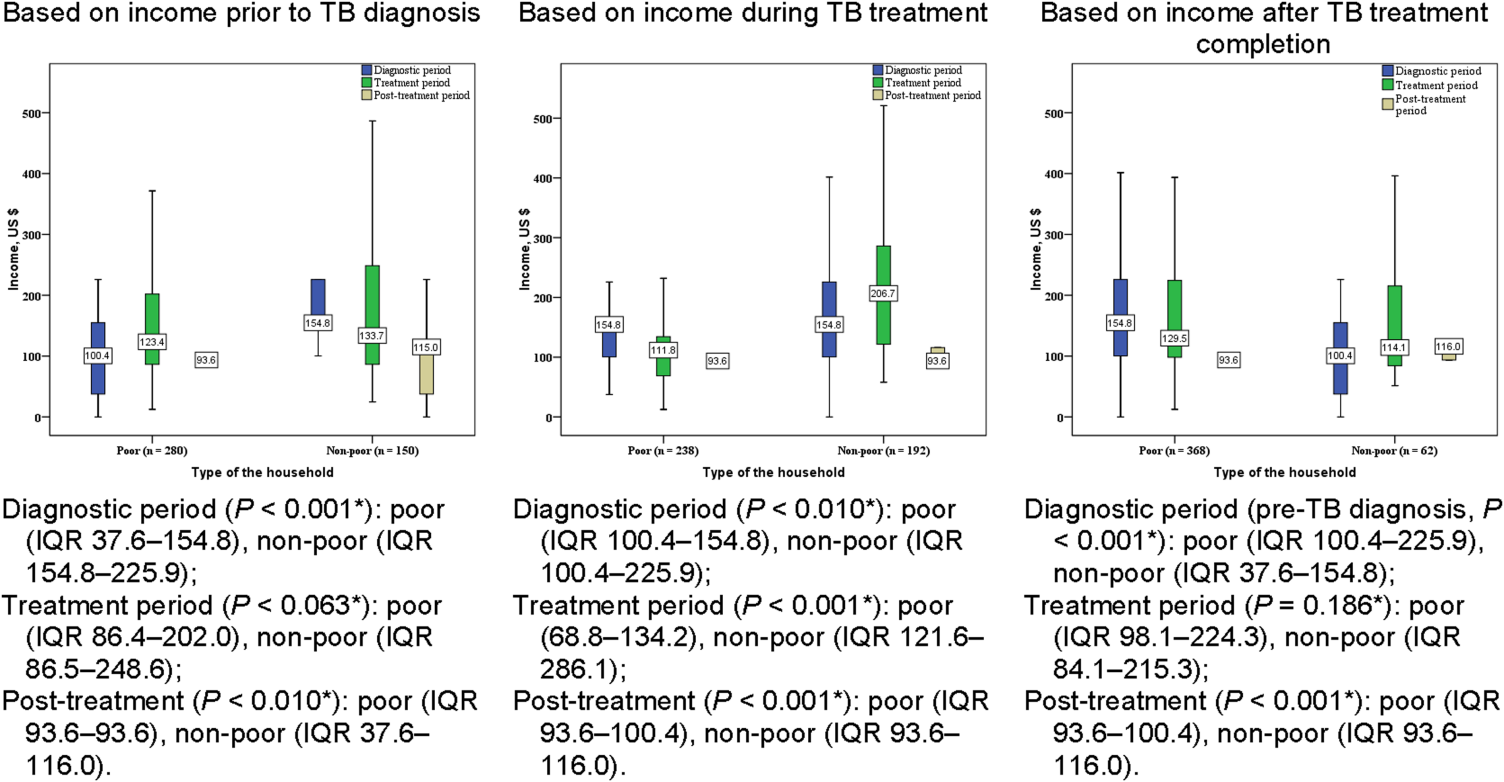
Median household income of poor (defined as household income ≤US$1.90 person/day) and non-poor (defined as household income >US$1.90 person/day) patients with RR-TB by TB treatment stage, Republic of Moldova, 2016. *Kruskal-Wallis test. RR-TB = rifampicin-resistant TB; IQR = interquartile range.

The median monthly income of TB-affected households who were classified as poor before TB diagnosis was US$100 (IQR 37–155), and this was US$94 (IQR 94–94) after treatment completion. There was a 1.4-fold decrease in median monthly income between pre-TB diagnosis and during TB treatment among those who were classified as poor at the TB treatment stage. TB-affected households who became poor after treatment completion had a median monthly income of US$94 (IQR 94–100), i.e., their income was 1.6-fold lower than that before TB (Figure 2).

Unadjusted analyses revealed several risks factors associated with poverty at each TB stage. TB patients with three or more people in their households were more likely to be poor before diagnosis (cOR 5.7, 95% CI 3.7–8.8), during treatment (cOR 3.8, 95% CI 2.5–5.6) and after treatment completion (cOR 7.2, 95% CI 3.6–14.3). Having a history of incarceration was a risk factor for poverty (cOR 2.3, 95% CI 1.1–5.2); however, being a labour migrant reduced the risk of poverty prior to TB (cOR 0.4, 95% CI 0.2–0.7). Those who were unofficially employed or unemployed were more likely to become poor during treatment (cOR 6.7, 95% CI 4.3–10.0). Being married or living with a partner was another risk factor for poverty after treatment completion (cOR 5.7, 95% CI 2.9–11.0) (Table).

**Figure 3.**
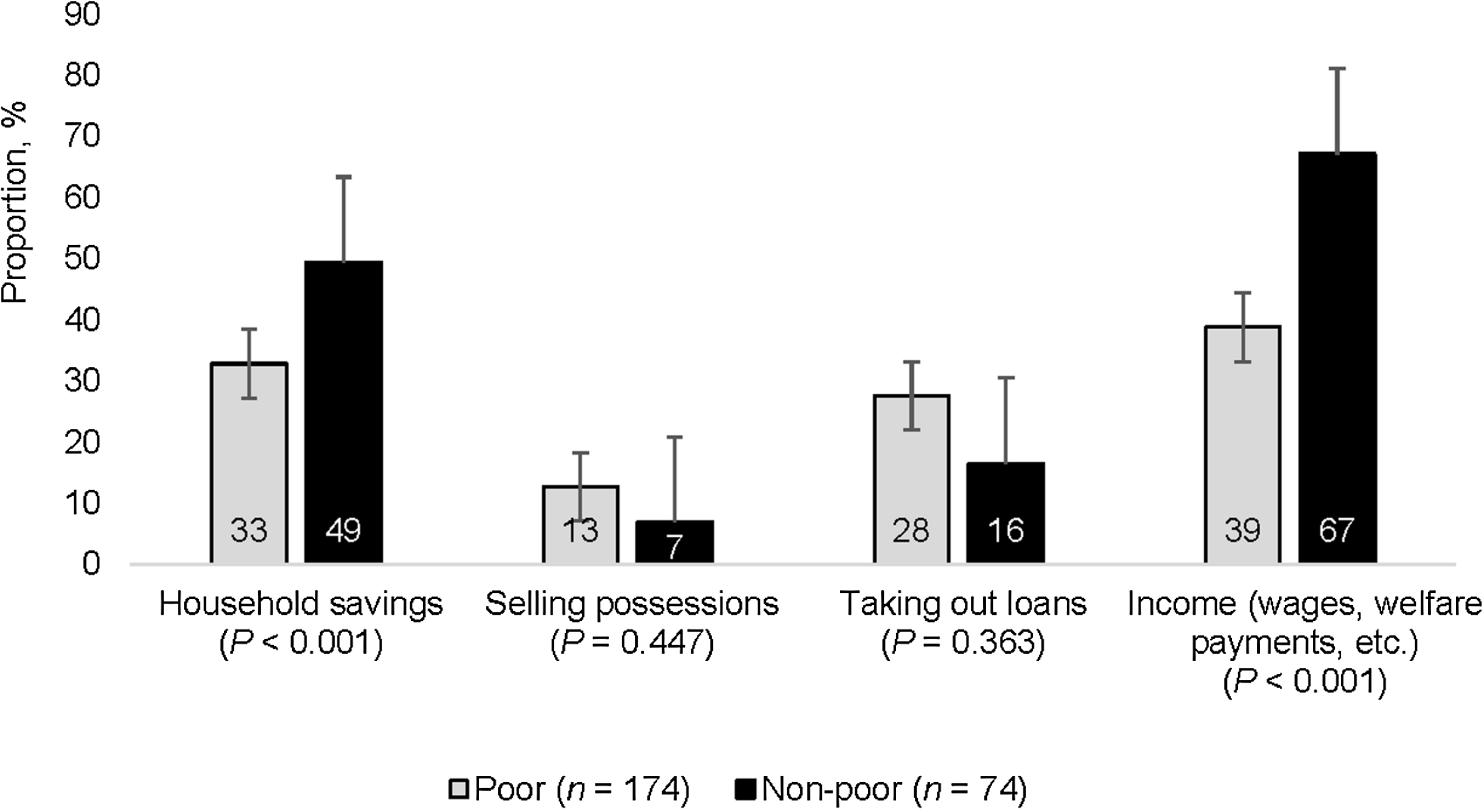
Sources of finance used to cover out-of-pocket payments in (defined as household income ≤US$1.90 person/day) and non-poor (defined as household income >US$1.90 person/day) households. Error bars represent 95% confidence intervals. χ^2^ tests used for proportions. *Based on household income prior to TB.

Approximately half (48%, 207/430) of TB patients experienced OOP expenditures between TB diagnosis and the end of TB treatment. Of these patients, 62% (174/280) were from poor households and 49% (74/150) were from non-poor households (*P* < 0.010). Both poor and non-poor TB patients had been obliged to use household savings, their income, to sell their possessions and to take out loans to cover OOP payments. Non-poor TB patients used savings and income to pay for OOP payments (*P* < 0.001; Figure 3).

## DISCUSSION

This study traces the patient’s journey through TB treatment and the relationship between TB treatment and poverty among patients in the Republic of Moldova in the WHO European Region. The results of this study confirm the relationship between TB and household poverty and present a few topics for discussion.

TB thrives on conditions of poverty and having TB can worsen poverty;^1^ however, poverty is difficult to measure as it is multifaceted. In this study, we measured economic poverty based on the World Bank criterion of poverty as people living on US$1.90 per person per day or less.^13^ The study showed that a high proportion of TB patients were poor before having TB, and economically poor people are at greater risk of TB infection than the general population.^1^ When we examined poverty during the different stages of TB (diagnostic, treatment and post-treatment), the proportion of participants who were poor during TB treatment decreased but increased after treatment completion. One explanation for the decrease in the proportion of poor patients during treatment is that patients with TB received welfare transfers (temporary allowances and adherence incentives) during treatment, which may have increased their household incomes. However, these temporary allowances are only given to patients with TB if they were officially employed before TB. During TB treatment, people who were not officially employed or who were not employed before TB were more likely to be living in poverty.

It has previously been shown that households can be driven into poverty once affected by TB, irrespective of the demographic or clinical characteristics,^15,16^ and this was also observed in this study. The main risk factors associated with poverty at each TB stage were social factors; for example, households with three or more members were more likely to be poor during all TB stages. This may be because low household incomes cannot cover the costs of the needs of each family member, especially if the household contains children and/or dependants. The average monthly income for poor households affected by TB was about three times lower than the average monthly income in the Republic of Moldova.^17^ Patients with TB who were married or cohabiting were likely to become poor after treatment completion due to an imbalance between the household income and the high expenses incurred during TB treatment that was observed among non-poor households. In addition, the original study^10^ showed that households experienced TB-related costs at the same time that their incomes were shrinking, thus creating a ‘medical poverty trap’.^18,19^

Having a history of incarceration was a risk factor for poverty before being infected with TB; both poverty and TB are common experiences for ex-prisoners. Previous incarceration is a known risk factors for latent TB infection (LTBI) and the development of active TB.^20^ Ex-prisoners often face barriers to employment and are more vulnerable to financial instability.

Being a labour migrant was found to be a protective factor for poverty before TB. In Moldova, government wages, social pensions and other unemployment benefits are small and, consequently, there are many people who are looking for informal work or who go to work abroad; 10% of the working age population is working or looking for a job abroad.^21^

The limitations of this study include the use of self-reported data for incomes of the respondents and their households, the use of estimation for the entire household income and the extrapolation of monthly reported incomes before TB treatment, during TB treatment and after treatment completion for all participants in the study.

## CONCLUSIONS

In the Republic of Moldova, TB mainly affects the poor and can drive RR-TB-affected households into poverty. In households affected by RR-TB, the proportion of poor households rose from 65% during the diagnostic period to 86% post-TB treatment. Several social factors that may have led to poverty were identified at each stage of TB, such as having a history of incarceration (during the diagnostic period); being unemployed or unofficially employed (during TB treatment); and being married or living with a partner (post-treatment). Having more than three members in a household was associated with poverty at all TB stages, and being a labour migrant was found to be a protective against poverty during the diagnostic period. To access TB services, patients spent their savings and incomes, and took loans and sold their possessions.

The results of this study highlight the role of social protection in TB prevention and indicate that innovative social protection policies are required to protect TB patients against poverty. A patient-centred approach and social protection interventions are essential for groups identified as being at-risk of TB.
